# Using homogenization, sonication and thermo-sonication to inactivate fungi

**DOI:** 10.7717/peerj.2020

**Published:** 2016-06-28

**Authors:** Daniela Campaniello, Antonio Bevilacqua, Milena Sinigaglia, Maria Rosaria Corbo

**Affiliations:** Department of the Science of Agriculture, Food and Environment, University of Foggia, Foggia, Italy

**Keywords:** Fungi, Desirability profile, Spores, Combination, Alternative approaches

## Abstract

Ultrasound (US), Thermo-sonication (TS) and High Pressure Homogenization (HPH) were studied as tools to inactivate the spores of *Penicillium* spp. and *Mucor* spp. inoculated in distilled water. For US, the power ranged from 40% to 100%, pulse from 2 to 10 s, and duration of the treatment from 2 to 10 min. TS was performed combining US (40–80% of power, for 8 min and pulse of 2 s) with a thermal treatment (50, 55 and 60°C at 4, 8 and 12 min). Homogenization was done at 30–150 MPa for 1, 2 and 3 times. Power was the most important factors to determine the antifungal effect of US and TS towards the conidia of *Penicillium* spp.; on the other hand, in US treatments *Mucor* spp. was also affected by pulse and time. HPH exerted a significant antifungal effect only if the highest pressures were applied for 2–3 times.

## Introduction

Thermal treatments are the most common techniques for preserving food products; however, heat causes vitamin loss, non-enzymatic browning, off flavors and odors; thus, the maintenance of the sensorial and freshness characteristics are not guaranteed. For these reasons, alternative processes have been introduced to replace traditional heat-processing (Rupasinghe & Yu, 2012). These methods are generally non-thermal approaches; they help retain nutritional and sensory quality while guaranteeing safety. Individually or combined with others, these techniques are able to reduce pathogens and spoilage microorganisms without negative effect on foods (Oliveira et al., 2015).

In this paper, we focused on three emerging technologies: Ultrasound (US), US combined with a heat treatment (Thermo-sonication; TS) and High Pressure Homogenization (HPH). US are pressure waves with frequencies of 20 kHz or more (Bevilacqua et al., 2014). The US effect relies upon cavitation: sonic wave encounters a liquid medium and creates longitudinal waves, generating regions of high and low pressure. These alternating pressures cause cavitation and gas bubble formation, increasing their volume until they implode. This event forms regions of high temperature and pressure exerting an antimicrobial effect, causing the breakdown of cell walls and inner membrane and the release of cell components (Di Benedetto, Perricone & Corbo, 2010; Klimek-Ochab et al., 2011).

Shape and dimension of microorganisms play an important role, thus microbial susceptibility can be resumed as follow: rods > cocci; larger cells > small cells; as well as Gram negative bacteria > Gram-positive bacteria. Spores are highly resistant to US and can be inactivated using combined treatments (Bevilacqua, Sinigaglia & Corbo, 2013). Compared to the single treatments, the combination of US with different techniques allows obtaining better results in terms of microbial inactivation (Aadil et al., 2015).

Heat combined with US (thermo-sonication) reduces process temperatures and processing times and leads to an improvement of the effects ensuring the inactivation of heat-resistant microorganisms and enzymes (Mason, Paniwnyk & Lorimer, 1996; Char et al., 2010). Heat and US act on the same target, although in a different way; heat causes a slight injury on cells and makes easier the breakage of cells and lack of cytoplasmic material by cavitation generated by ultrasound (Bermúdez-Aguirre, Mawson & Barbosa-Cánovas, 2011); type, morphology or diameter of microorganisms also affect thermo-sonication efficiency.

High Pressure Homogenization (HPH) is a technique based on the application of continuous or semi-continuous pressures between 60 and 400 MPa. It is generally applied to reduce the particles to finer dimensions: fluid passes in the homogenizing valve creating conditions of high turbulence and shear, combined with compression, acceleration, sudden pressure changes and impact. Thus, particles appear disintegrated and dispersed throughout the product; this comminution effect of HPH also causes the disruption of the cell membranes of microorganisms. The microbial inactivation depends on pressure, temperature, number of passes and type of medium and microorganisms. Literature reports that HPH can inactivate various types of bacteria (Diels & Michiels, 2006; Bevilacqua et al., 2009); some data are also available for fungi (Corbo et al., 2010).

It is well known that moulds are thermo-resistant even in foods pasteurized and packed; the presence of spores allow fungi to survive thermal treatment thus leading to the deterioration of foods and, in many cases, to risks for the consumers. Furthermore, the production of toxins is a serious hazard for food safety (Ferreira et al., 2011). Thus, this work was aimed to study three physical treatments (US, TS and HPH) as tools to inactivate the spores of *Mucor* spp. and *Penicillium* spp. *Mucor* species (for example, *Mucor circinelloides*), isolated from yogurt, cause mucormycosis and food-borne illness (Lee et al., 2014). The antifungal effect of the three treatments was studied by using the theory of the design of the experiments to pinpoint the effect of each variable (power, pulse and duration of the treatment for US; pressure level for HPH; combination of thermal treatments and sonication for TS), as well the effect of storage after the treatment.

## Materials and Methods

### Fungal spore production

This research focused on two fungi (*Penicillium* spp., and *Mucor* spp.) belonging to the Culture Collection of the laboratory of Predictive Microbiology, University of Foggia. The strains were isolates from soil; the identification was performed based on the common phenotypic traits of fungi used for taxonomic purposes.

The moulds were grown on Potato Dextrose Agar plates (PDA) (Oxoid, Milan, Italy), and incubated at 25°C for 1 week. A spore suspension of each strain was prepared by washing the mould on PDA plates with a Tween 80 solution (0.05% v/v) (C. Erba, Milan, Italy), as described by Sinigaglia, Corbo & Ciccarone (1998). Conidia concentration after the preparation was ca. 10^7^ cfu/mL, evaluated through the spread plate method on PDA plates (incubated at 25°C for 5 days). Each suspension was filtered to avoid the presence of mycelium. Conidia suspension was used immediately.

### Ultrasound

Aliquots of 20 mL of distilled water were inoculated to ca. 10^7^ cfu/mL with *Penicillium* spp. and *Mucor* spp., separately. Thereafter, the samples were treated by a Vibra Cell Ultrasound equipment, model VC 130 (Sonics and Materials Inc., Newtown, CT, USA); the equipment works at 20 kHz (frequency)-130 W (acoustic energy). The probe (5 × 60 mm; diameter × the active component of horn) was put 2–3 cm below the surface of the samples; power level, duration of the treatment and pulse were combined using a randomized design (Table 1). The mean transducer efficiency of probe was 70% (*η*); the effective acoustic power or energy supplied (WA) into the media can be evaluated as follows:

\begin{eqnarray*}WA={\eta }^{\ast }Pe. \end{eqnarray*}

Pe being power input (130 W). Thus, the net energy supplied by the transducer varied from 36.4 W (40% of total power applied) to 91 W (100% of acoustic energy).

**Table 1:** Randomized design of US process.

Samples	Power (%)	Duration of the treatment (min)	Pulse (s)
A	40	2	2
B	40	2	10
C	40	6	2
D	40	6	10
E	40	10	2
F	40	10	10
G	60	2	2
H	60	2	10
I	60	6	2
L	60	6	10
M	100	2	2
N	100	2	10
O	100	6	2
P	100	6	10
Control	–	–	–

Before each treatment, the ultrasonic probe was cleaned with ethanol and washed with sterile distilled water. Just after processing, samples were cooled in ice and water; the exit temperature before cooling was 60–65°C (for the sample processed for 10 min).

### Thermo-sonication

Aliquots of 20 mL of distilled water were inoculated to ca. 10^7^ cfu/mL with *Penicillium* spp. and *Mucor*spp., separately. TS was performed by using a water bath (50, 55 and 60°C for 4, 8 and 12 min) and a US equipment (power, 40–80%; pulse, 2 s; time, 8 min). Two assays were done: test A (thermal treatment, TT, followed by US) and test B (US followed by TT). Power, temperature and duration of the thermal treatment were combined using a 3^*k*−*p*^ design. Tables 2 and 3 show coded and real values of independent variables and their combinations, respectively.

**Table 2:** Coded values and real values of independent variables for TS processing.

Independent variables	Coded levels		

	0	0.5	1
Temperature (°C)	50	55	60
Duration of thermal treatment (min)	4	8	12
US-power (%)	40	60	80

**Table 3:** Combination of the design for TS processing. These combinations were used for both assay A and assay B.

Samples	Temperature (°C)	Duration of the thermal treatment (min)	Power (%)
A	60	4	40
B	50	12	40
C	50	4	80
D	55	8	80
E	55	4	60
F	50	8	60
G	Control	–	–
H	–	–	80
I	60	4	–
L	60	12	–

### High pressure homogenization

Aliquots of 1 L of distilled water were inoculated to ca. 10^7^ cfu/mL with *Penicillium* spp. and *Mucor*spp., separately; then, all the samples were homogenized as follows.

(a) Single step processing

Samples were homogenized through a high-pressure homogenizer (PANDA 2 K homogenizer, Niro Soavi s.p.a., Parma, Italy) at pressure levels between 30 and 150 MPa. The exit temperature of the samples was ca. 45–50°C at 150 MPa; thus, they were immediately cooled to 20°C in a water bath. The circuits of the equipment were cleaned with hot sterile and distilled water (70°C).

(b) Multi-step processing

Samples were homogenized 2 or 3 times at 120 and 150 MPa. After each pass the samples were immediately cooled, as reported above; the rest time amongst the different passes was ca. 5 min.

The samples (both those from single and multi-step) were stored at 25°C for 14 days.

### Microbial analyses

The number of surviving conidia was evaluated through the spread plate method on PDA incubated at 25°C for 5 days immediately after the treatment and for at least 14 days. Aliquots of distilled water, inoculated with fungi but not treated through US, TS and HPH were used as controls.

### Statistical analysis

All the experiments were performed at least over two independent batches. The experiments were performed twice.

#### US and TS

Data were modelled as a decrease of population referred to the control (Nc–Ns, where Nc and Ns are the levels of mould in the control and the test samples, respectively) (US) or as level of conidia (TS), submitted to a multiple regression procedure through the software Statistica for Windows 10.0 (Statsoft, Tulsa, OK).

A Pareto chart of the standardized effects was used to point out if a variable was significant or not; the standardized effects were evaluated as the ratio of the mathematical coefficient of each term of the equation vs its standard error. If the effect overcame the significance breakpoint (*P* < 0.05), corresponding to the vertical line, it was regarded as significant and included into the equation. The 3D-ternary plots show the correlation of the dependent variables with the independent ones.

In addition, the effect of each independent variable on the decrease of cell count was evaluated through the individual desirability functions, estimated as follows:

\begin{eqnarray*}d= \left\{ \begin{array}{@{}ll@{}} \displaystyle 0,&\displaystyle y\leq {y}_{min}\\ \displaystyle (y-{y}_{min})/({y}_{max}-{y}_{min})&\displaystyle {y}_{min}\leq y\leq {y}_{max}\\ \displaystyle 1,&\displaystyle y\geq {y}_{max} \end{array} \right. \end{eqnarray*}

where ${y}_{\mathrm{min}}$ and ${y}_{\mathrm{max}}$ are the minimum and maximum values of the dependent variable, respectively.

#### HPH

Data were analyzed through one-way analysis of variance and Tukey’s test (*P* < 0.05).

## Results and Discussion

### Sonication

**Figure 1:**
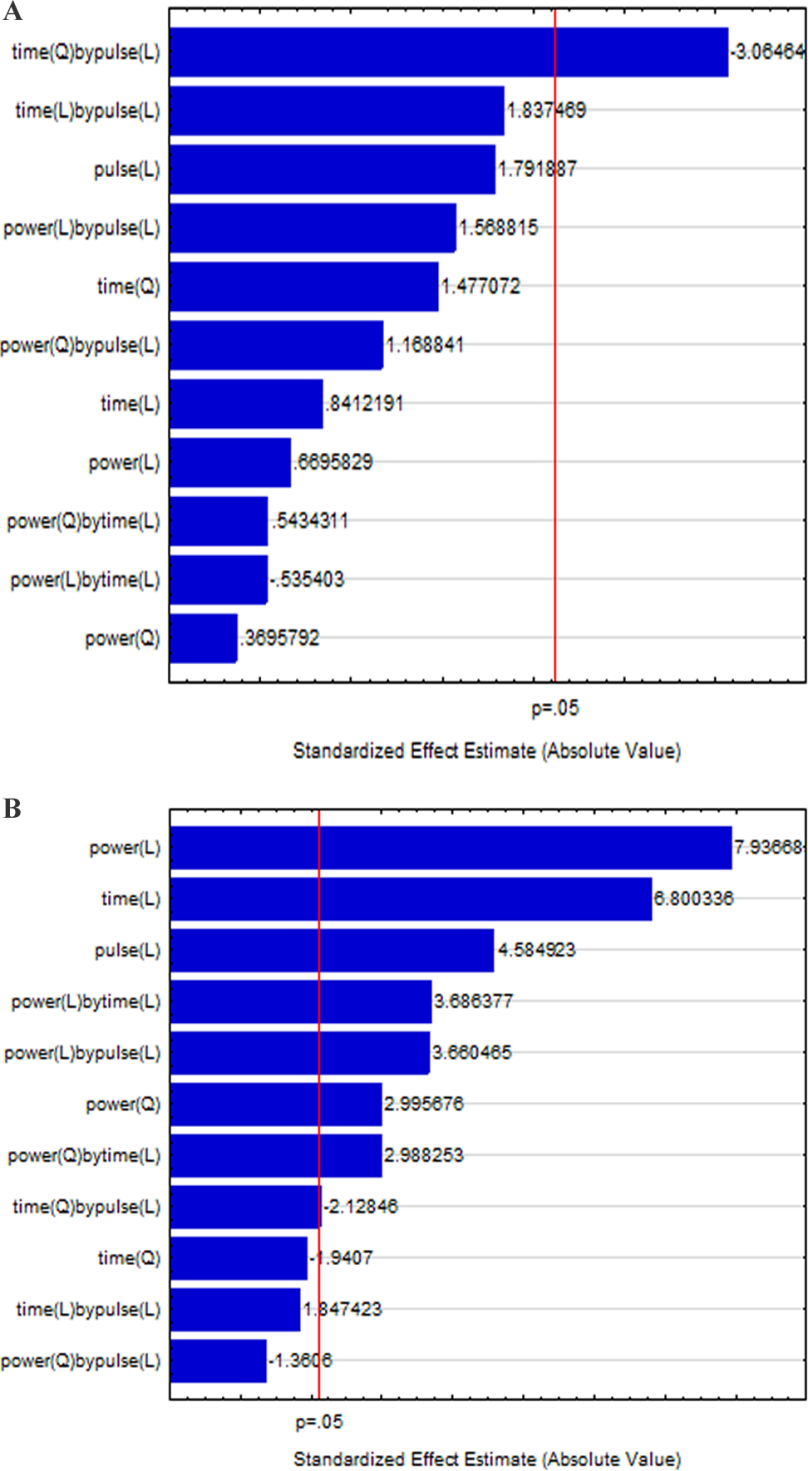
Pareto chart of the standardized effects of power, pulse and duration (time) of US processing the reduction of the spores of *Penicillium* spp. (A) and *Mucor* spp. (B) in distilled water immediately after the treatment. L, linear effect; Q, quadratic effect.

Figure 1A and 1B shows the Pareto chart of standardized effects of power, pulse and time on the antifungal activity of US on *Penicillium* spp. and *Mucor* spp. immediately after the treatment.

*Penicillium* was affected only by the interaction [time] × [pulse]; all the other effects were not significant (Fig. 1A). Otherwise, *Mucor* spp. was affected by the linear terms of power, pulse and time, as well as by some interactive terms and by the quadratic term of power (Fig. 1B).

**Figure 2:**
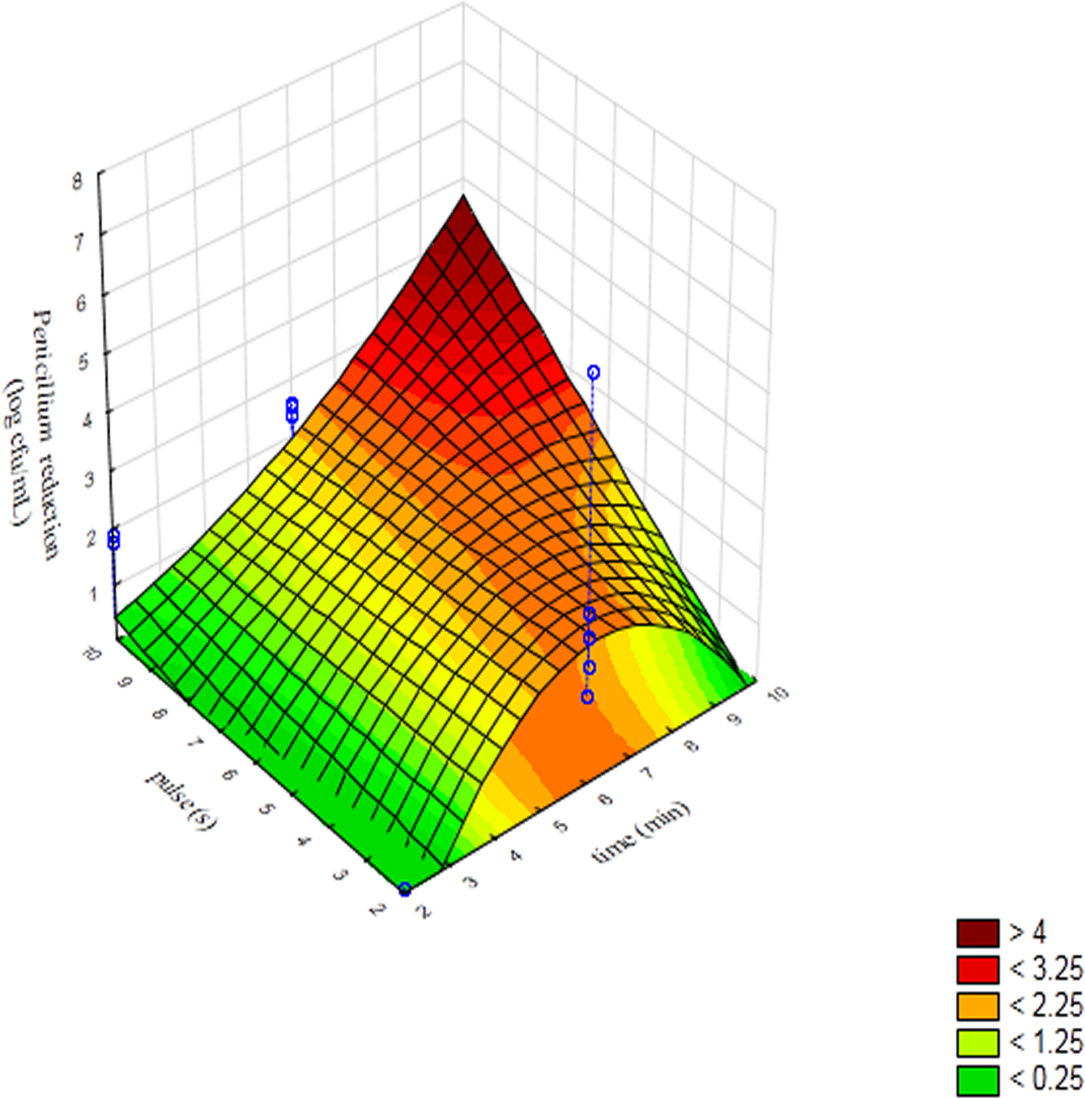
Surface response plot for the interaction [time] × [pulse] on the reduction of the spores of *Penicillium* immediately after sonication.

**Figure 3:**
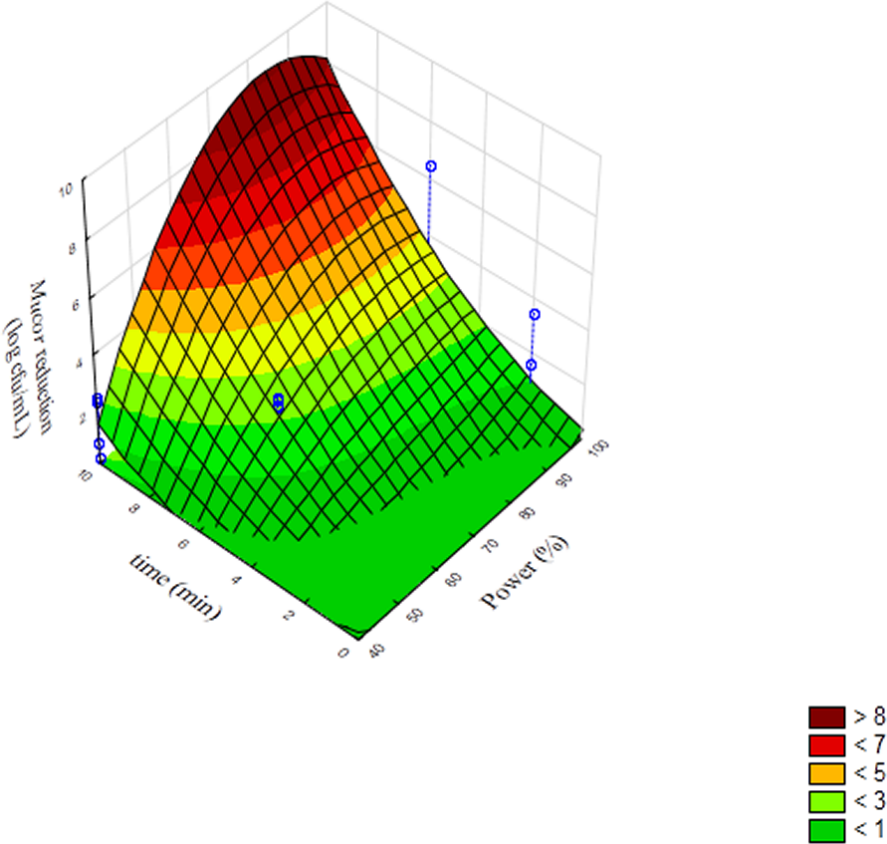
Surface response plot for the interaction [time] × [power] on the reduction of the spores of *Mucor* immediately after sonication.

Pareto chart is a qualitative output, i.e., it can highlight if a term is significant or not but it cannot point out how much a term is significant; this information could be obtained through the 3D-plots. Figure 2 reports the surface response for the interaction [time] × [pulse] on the reduction of *Penicillium* spores. A reduction of 4 log cfu/mL was found for a treatment of 10 min and pulse set to 10 s. Concerning *Mucor*, the model predicted a spore reduction of 8 log cfu/mL in correspondence of 80% of power and after 8 or 10 min of treatment (Fig. 3); this is obviously a prediction, as we inoculated ca. 7 log cfu/mL. This higher value pinpointed the strong effect of the factors on the performance of the treatment and the reduction of the target below the detection limit.

Interesting results (spore reduction of ca. 5 cfu/mL) were also obtained after 6 min. The 3D plots are useful tools; however, they focus on the interaction between two parameters and many times could mask the effect of a single hurdle. An alternative approach to focus on the effect of each factor is a desirability profile. Generally, a desirability profile contains two pictures for each factor: the predicted values and desirability outputs (from 0 to 1).

**Figure 4:**
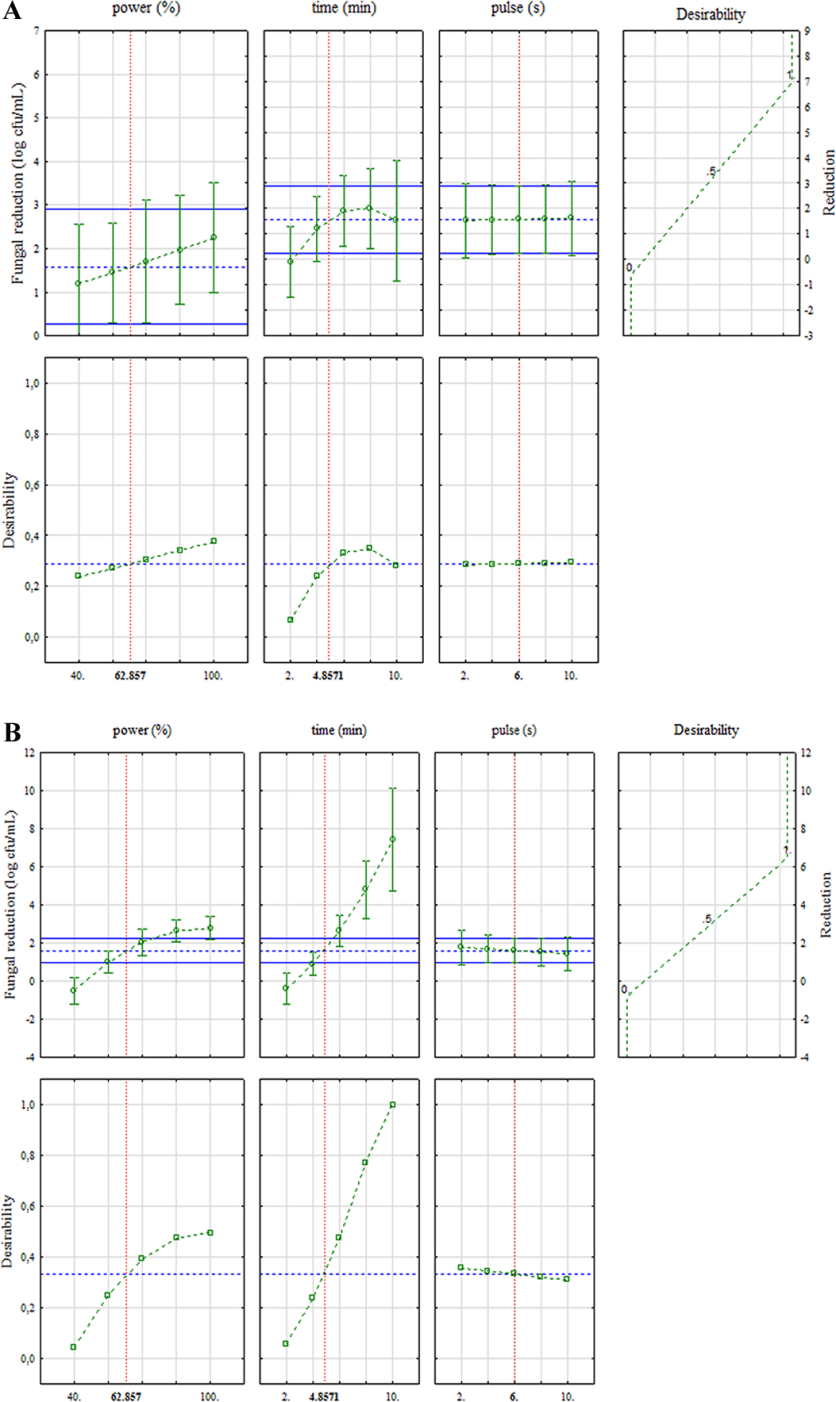
Desirability profile for the effects of power, duration of the treatment and pulse on the survival (decrease of spore number referred to control) of *Penicillium* spp. (A) and *Mucor* spp. (B).

Figure 4A is a desirability profile for *Penicillium*; it shows some differences if compared to the Pareto chart. In fact, the profile pinpointed a positive trend of power, a not linear effect of time (antifungal effect of US increases as time increased up to a critical threshold of 6–7.5 min) and a slight effect of pulse. This difference could be due to the fact that probably the statistical weight of interaction time/pulse was strong and masked the effect of power. Concerning *Mucor*, the desirability profile confirmed the stronger effect of power and time (Fig. 4B).

Many papers focused on the antimicrobial activity of US but only few researchers studied US antifungal activity (López-Malo et al., 2005; Bevilacqua, Sinigaglia & Corbo, 2013) and there is a debate on which is the most important variable for the antifungal activity of US. Bilek & Turantaş (2013) reported that the antimicrobial efficiency of ultrasound is relatively low and that US could be used as an alternative method only under special conditions. It is known that temperature, amplitude of the ultrasonic waves, duration of the treatment (time), type of microorganism, volume and composition of matrix, act differently on the antimicrobial effectiveness of US (Ganesan et al., 2015); thus, the optimal combination of these ones becomes necessary to obtain the best performance of the US treatment. Bevilacqua et al. (2015) combined power, duration of the treatment and pulse through a randomized fractional design to evaluate US effectiveness towards *Pichia* spp. and *Wickerhamomyces anomalus* and found that power was the most significant parameter. On the other hand, Sainz Herrán et al. ( 2010) studied the effects of US on *Aspergillus terreus* and observed that US affected fungal morphology but not the growth rate at any power.

Bevilacqua, Sinigaglia & Corbo (2013) reported that *Fusarium oxysporum* was strongly influenced by the interactive effect of power, time and pulse, suggesting a critical role of the total process energy. Our data confirm these hypothesis, although the effect on *Penicillium* spp. was more pronounced than on *Mucor* spp.

### Thermo-sonication

Heat combined with ultrasound was assessed as a promising tool to find a possible synergy; thus, two assays were performed: test A (TT and US) and test B (US and TT).

Table 4 reports the statistical effects of the thermo-sonication parameters (for both the assay A and B); a multiple regression approach was used to fit the data, thus the table shows the mathematical coefficients for single, quadratic and interactive terms. Some terms were not significant, whereas significant factors were generally negative. A negative term pinpoints an inverse correlation of the factors vs spore level, i.e., an increase of the intensity of the factor caused a significant decrease of spore number of *Penicillium* and *Mucor*.

**Table 4:** Impact of temperature and sonication power on the level of fungal spores of *Penicillium* spp. and *Mucor* spp. immediately after the treatment. Assay A: thermal treatment and sonication; assay B: sonication and thermal treatment.

	*Penicillium* spp.		*Mucor* spp.	

	A	B	A	B
**Individual effect**				
Power	−0.063^*^	−0.103	–	–
Temperature	–^**^	–	−0.089	–
**Interaction**				
Power*temperature	–	–	–	−0.001
**Quadratic effects**				
Power^2^	–	–	–	0.001
Temperature^2^	–	–	–	−0.002
**Significance of the model**				
R	0.732	0.773	0.784	0.849
ES	1.616	1.422	1.578	1.148

**Notes.**

* Mathematical coefficient.

** Not significant.

**Figure 5:**
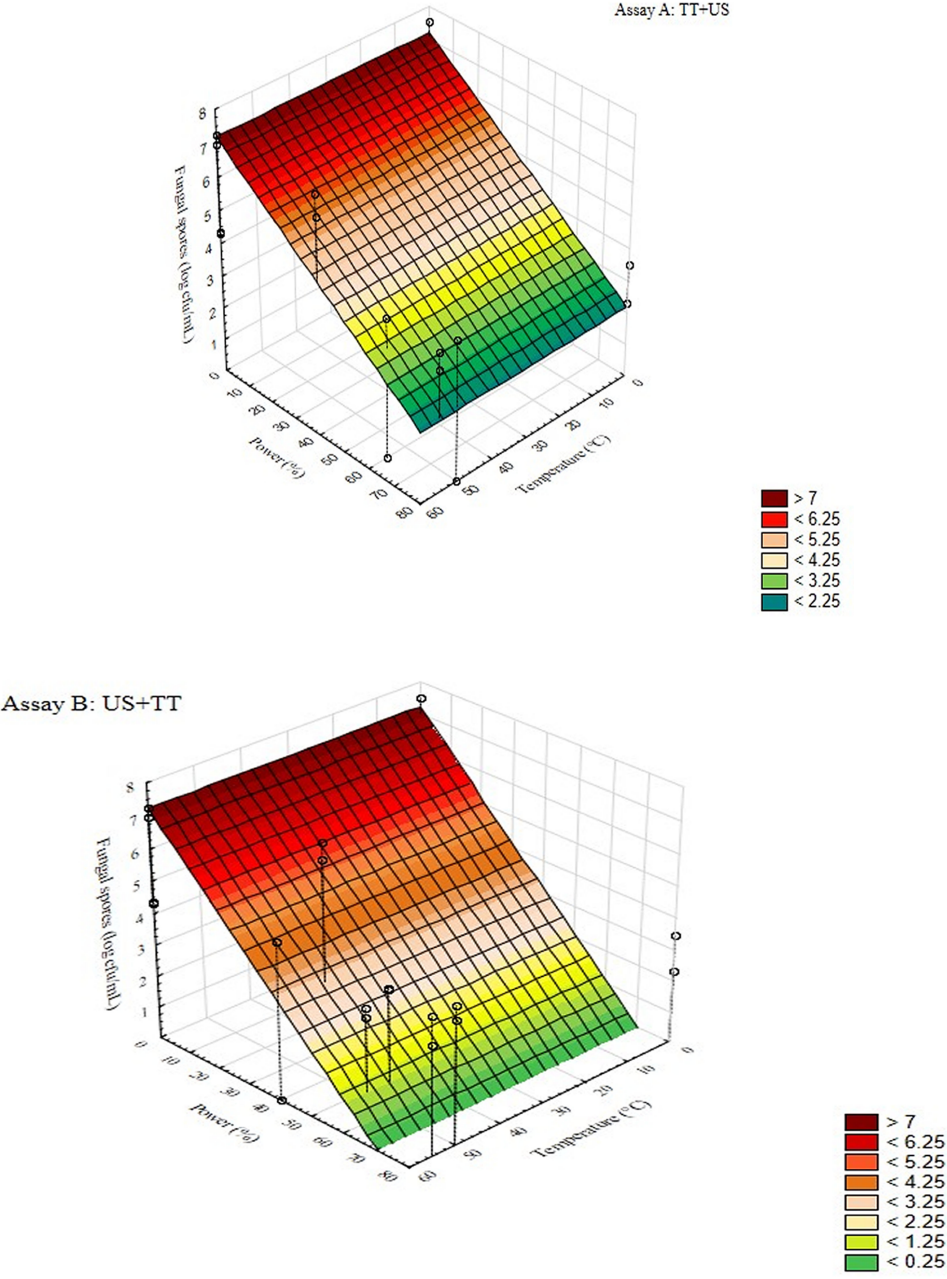
3D plots for the interaction [power] × [temperature] for the assay A and B, of thermo-sonication on *Penicillium* spp.

*Penicillium* spp. was always affected by power both in assay A and B; on the other hand, the impact of sonication and thermal treatment on *Mucor* spp. relied upon the kind of combination. In fact, in assay A (thermal treatment + sonication), the temperature of thermal treatment was the only factor playing a role in the inactivation of the target mould and sonication was not significant. The net power of sonication played a significant role as quadratic term or in interaction with temperature only in assay B (sonication + thermal treatment).

Figure 5A reports 3D plots for the interaction [power] ×[temperature] for the assay A on *Penicillium* spp. The figure highlights the role of sonication on mould inactivation and shows that an increase of the power up to 80% reduced spore level from 7 to 2.25 log cfu/mL. Similar results were found for the assay B, although the slope of the surface plot was different and at 80% the spores of *Penicillium* were below the detection limit (1 log cfu/mL) (Fig. 5B).

We can assume that as US causes the thinning of the cell membranes (Di Benedetto, Perricone & Corbo, 2010), thus it significantly weakens fungal strain. Furthermore, US causes a localized heating which could lead to an additional weakness of *Penicillium* spp.

To date, literature is limited in studies concerning the antifungal effectiveness of Thermo-sonication. López-Malo et al. (2005) demonstrated that thermo-sonication combined with antimicrobial compounds (vanillin and potassium sorbate) inhibited *Aspergillus flavus* and *Penicillium digitatum* survival.

As reported by Leistner (1999) it is difficult to obtain complete microbial inactivation through single treatments. According to this concept, the combination of US with heat performed in this paper proved to be an effective treatment and provided encouraging results.

### Homogenization

Table 5 reports *Penicillium* spp. in distilled water stored at 25°C for 14 days. A single-step process did not reduce fungal spores; a significant effect was found after 2–3 steps at 150 MPa. *Penicillium* spp. was reduced from 7.6 to 6.5 cfu/mL after 2 and 3 passes immediately after the treatment; moreover spores were reduced below the detection limit after 7 (3 steps) or 14 days (2 steps).

The effect of HPH towards *Mucor* spp. was slight at 120 MPa with a 0.8 log cfu/mL reduction (from 6.6 log cfu/mL to 5.8 log cfu/mL) while at 150 MPa conidia were reduced by 1.2 log cfu/mL (from 6.6 log cfu/mL to 5.4 log cfu/mL). Then, *Mucor* spp. was completely inactivated throughout storage (Table 6). Finally *Mucor* spp. also experienced a complete inhibition at 120 MPa-3 steps and 150 MPa-2 and 3 steps.

**Table 5:** *Penicillium* spp. (log cfu/mL) in distilled water stored at 25 °C for 14 days after homogenization. Mean values ± SD.

Samples (MPa-no of passes)	Time (days)			

	0	2	7	14
A (30 –1)	7.62 ± 0.353${{\text{}}^{\mathrm{a}}}_{\mathrm{A}}$	7.51 ± 0.206${{\text{}}^{\mathrm{a}}}_{\mathrm{A}}$	7.49 ± 0.234${{\text{}}^{\mathrm{a}}}_{\mathrm{A}}$	7.11 ± 0.162${{\text{}}^{\mathrm{a}}}_{\mathrm{A}}$
B (60 –1)	7.32 ± 0.000${{\text{}}^{\mathrm{a}}}_{\mathrm{A}}$	7.57 ± 0.024${{\text{}}^{\mathrm{a}}}_{\mathrm{A}}$	7.21 ± 0.095${{\text{}}^{\mathrm{a}}}_{\mathrm{A}}$	7.41 ± 0.095${{\text{}}^{\mathrm{a}}}_{\mathrm{A}}$
C (90 –1)	7.30 ± 0.010${{\text{}}^{\mathrm{a}}}_{\mathrm{A}}$	7.48 ± 0.255${{\text{}}^{\mathrm{a}}}_{\mathrm{A}}$	7.36 ± 0.345${{\text{}}^{\mathrm{a}}}_{\mathrm{A}}$	7.32 ± 0.396${{\text{}}^{\mathrm{a}}}_{\mathrm{A}}$
D (120 –1)	7.56 ± 0.022${{\text{}}^{\mathrm{a}}}_{\mathrm{A}}$	7.52 ± 0.310${{\text{}}^{\mathrm{a}}}_{\mathrm{A}}$	7.19 ± 0.243${{\text{}}^{\mathrm{a}}}_{\mathrm{A}}$	7.65 ± 0.068${{\text{}}^{\mathrm{a}}}_{\mathrm{A}}$
E (120 –2)	7.56 ± 0.141${{\text{}}^{\mathrm{a}}}_{\mathrm{A}}$	7.32 ± 0.337${{\text{}}^{\mathrm{a}}}_{\mathrm{A}}$	7.11 ± 0.162${{\text{}}^{\mathrm{a}}}_{\mathrm{A}}$	7.39 ± 0.124${{\text{}}^{\mathrm{a}}}_{\mathrm{A}}$
F (120 –3)	7.30 ± 0.091${{\text{}}^{\mathrm{a}}}_{\mathrm{A}}$	7.37 ± 0.831${{\text{}}^{\mathrm{a}}}_{\mathrm{A}}$	7.12 ± 0.597${{\text{}}^{\mathrm{a}}}_{\mathrm{A}}$	6.30 ± 0.000${{\text{}}^{\mathrm{b}}}_{\mathrm{A}}$
G (150 –1)	7.60 ± 0.073${{\text{}}^{\mathrm{a}}}_{\mathrm{A}}$	7.68 ± 0.026${{\text{}}^{\mathrm{a}}}_{\mathrm{A}}$	7.07 ± 0.103${{\text{}}^{\mathrm{a}}}_{\mathrm{A}}$	7.37 ± 0.323${{\text{}}^{\mathrm{a}}}_{\mathrm{A}}$
H (150 –2)	6.30 ± 0.000${{\text{}}^{\mathrm{b}}}_{\mathrm{A}}$	6.48 ± 0.000${{\text{}}^{\mathrm{b}}}_{\mathrm{A}}$	6.00 ± 0.010${{\text{}}^{\mathrm{b}}}_{\mathrm{A}}$	–${{\text{}}^{\mathrm{c}}}_{\mathrm{B}}$
I (150 –3)	6.48 ± 0.040${{\text{}}^{\mathrm{b}}}_{\mathrm{A}}$	5.50 ± 0.707${{\text{}}^{\mathrm{c}}}_{\mathrm{B}}$	–${{\text{}}^{\mathrm{c}}}_{\mathrm{C}}$	–${{\text{}}^{\mathrm{c}}}_{\mathrm{C}}$
Control	7.61 ± 0.000${{\text{}}^{\mathrm{a}}}_{\mathrm{A}}$	7.71 ± 0.275${{\text{}}^{\mathrm{a}}}_{\mathrm{A}}$	7.39 ± 0.265${{\text{}}^{\mathrm{a}}}_{\mathrm{A}}$	7.80 ± 0.033${{\text{}}^{\mathrm{a}}}_{\mathrm{A}}$

**Notes.**

${\text{}}^{\mathrm{A}}$For each row values with different capital letters are significantly different (one-way ANOVA and Tukey’s test) (*P* < 0.05).

${\text{}}^{\mathrm{a}}$For each column values with different small letters are significantly different (one-way ANOVA and Tukey’s test) (*P* < 0.05).

^−^Surviving conidia below the detection limit (log cfu/mL).

**Table 6:** *Mucor* spp. (log cfu/mL) in distilled water stored at 25 °C for 14 days after homogenization. Mean values ± SD.

Samples (MPa-no of the passes)	Time (days)			

	0	2	7	14
A (30 –1)	6.59 ± 0.156${{\text{}}^{\mathrm{a}}}_{\mathrm{A}}$	6.84 ± 0.088${{\text{}}^{\mathrm{a}}}_{\mathrm{A}}$	6.54 ± 0.337${{\text{}}^{\mathrm{a}}}_{\mathrm{A}}$	6.50 ± 0.281${{\text{}}^{\mathrm{a}}}_{\mathrm{A}}$
B (60 –1)	6.65 ± 0.068${{\text{}}^{\mathrm{a}}}_{\mathrm{A}}$	6.63 ± 0.212${{\text{}}^{\mathrm{a}}}_{\mathrm{A}}$	6.31 ± 0.659${{\text{}}^{\mathrm{a}}}_{\mathrm{A}}$	6.57 ± 0.384${{\text{}}^{\mathrm{a}}}_{\mathrm{A}}$
C (90 –1)	6.50 ± 0.281${{\text{}}^{\mathrm{a}}}_{\mathrm{A}}$	6.59 ± 0.156${{\text{}}^{\mathrm{a}}}_{\mathrm{A}}$	6.10 ± 0.707${{\text{}}^{\mathrm{a}}}_{\mathrm{A}}$	5.90 ± 0.831${{\text{}}^{\mathrm{a,b}}}_{\mathrm{A}}$
D (120 –1)	5.83 ± 0.180${{\text{}}^{\mathrm{a,b}}}_{\mathrm{A}}$	5.90 ± 0.077${{\text{}}^{\mathrm{b}}}_{\mathrm{A}}$	6.24 ± 0.337${{\text{}}^{\mathrm{a}}}_{\mathrm{B}}$	6.39 ± 0.124${{\text{}}^{\mathrm{a}}}_{\mathrm{A}}$
E (120 –2)	5.60 ± 0.000${{\text{}}^{\mathrm{b}}}_{\mathrm{A}}$	5.54 ± 0.088${{\text{}}^{\mathrm{b}}}_{\mathrm{A}}$	–${{\text{}}^{\mathrm{b}}}_{\mathrm{B}}$	–${{\text{}}^{\mathrm{c}}}_{\mathrm{B}}$
F (120 –3)	–${{\text{}}^{\mathrm{c}}}_{\mathrm{A}}$	–${{\text{}}^{\mathrm{c}}}_{\mathrm{A}}$	–${{\text{}}^{\mathrm{b}}}_{\mathrm{A}}$	–${{\text{}}^{\mathrm{c}}}_{\mathrm{A}}$
G (150 –1)	5.40 ± 0.033${{\text{}}^{\mathrm{b}}}_{\mathrm{A}}$	5.48 ± 0.016${{\text{}}^{\mathrm{b}}}_{\mathrm{A}}$	–${{\text{}}^{\mathrm{b}}}_{\mathrm{A}}$	–${{\text{}}^{\mathrm{c}}}_{\mathrm{A}}$
H (150 –2)	–${{\text{}}^{\mathrm{c}}}_{\mathrm{A}}$	–${{\text{}}^{\mathrm{c}}}_{\mathrm{A}}$	–${{\text{}}^{\mathrm{b}}}_{\mathrm{A}}$	–${{\text{}}^{\mathrm{c}}}_{\mathrm{A}}$
I (150 –3)	–${{\text{}}^{\mathrm{c}}}_{\mathrm{A}}$	–${{\text{}}^{\mathrm{c}}}_{\mathrm{A}}$	–${{\text{}}^{\mathrm{b}}}_{\mathrm{A}}$	–${{\text{}}^{\mathrm{c}}}_{\mathrm{A}}$
Control	6.63 ± 0.212${{\text{}}^{\mathrm{a}}}_{\mathrm{A}}$	6.77 ± 0.103${{\text{}}^{\mathrm{a}}}_{\mathrm{A}}$	6.82 ± 0.031${{\text{}}^{\mathrm{a}}}_{\mathrm{A}}$	6.54 ± 0.088${{\text{}}^{\mathrm{a}}}_{\mathrm{A}}$

**Notes.**

${\text{}}^{\mathrm{A}}$For each row values with different capital letters are significantly different (one-way ANOVA and Tukey’s test) (*P* < 0.05).

${\text{}}^{\mathrm{a}}$For each column values with different small letters are significantly different (one-way ANOVA and Tukey’s test) (*P* < 0.05).

^−^Surviving conidia below the detection limit (log cfu/mL).

HPH represents a very interesting treatment (Diels & Michiels, 2006; Wuytack, Diels & Michiels, 2002; Patrignani et al., 2010; Bevilacqua et al., 2009), but literature is limited for the effect of this approach towards moulds. Only few authors have recently addressed this topic. Corbo et al. (2010) studied the use of HPH to control the growth of *Fusarium oxysporum*, *Emericella nidulans* and *Penicillium italicum* highlighting that increasing the pressure and the number of the passes determined a significant effect on conidia.

Our results are in agreement with Corbo et al. (2010) as the efficiency of the treatment on *Penicillium* spp. and *Mucor* spp. was evident at higher pressures and during a multi-step HPH.

Accordingly, our research confirmed that homogenization alone could not be used to inactivate fungal spores and that its combination with some antimicrobials would be advisable, as suggested by Bevilacqua et al. (2012).

## Conclusions

There is an increasing interest towards the use of non-thermal technologies for the inactivation of foodborne spoilers. This paper focused on the antifungal effect of homogenization, ultrasound, and thermo-sonication on two different moulds. Ultrasound could be successfully used to inactivate *Penicillium* spp. and *Mucor* spp. and its effects mainly relied upon power; a slight thermal treatment could be used to increase the effectiveness of sonication on *Mucor* but not on *Penicillium*.

Concerning HPH, the effectiveness of the process was influenced by pressure levels and number of passes; *Penicillium* spp. and *Mucor* spp. were inhibited throughout storage, at the highest pressures and after 2 or 3 passes. Although this paper provided encouraging results, further investigations are required to validate these data *in vivo* and towards a wide range of fungi.

##  Supplemental Information

10.7717/peerj.2020/supp-1 Data S1 Data set of US, TS and HPH Log ufc/ml of Penicillium and Mucor treated

## Additional Information and Declarations

### Data Availability

The following information was supplied regarding data availability: The raw data has been supplied as Data S1.
